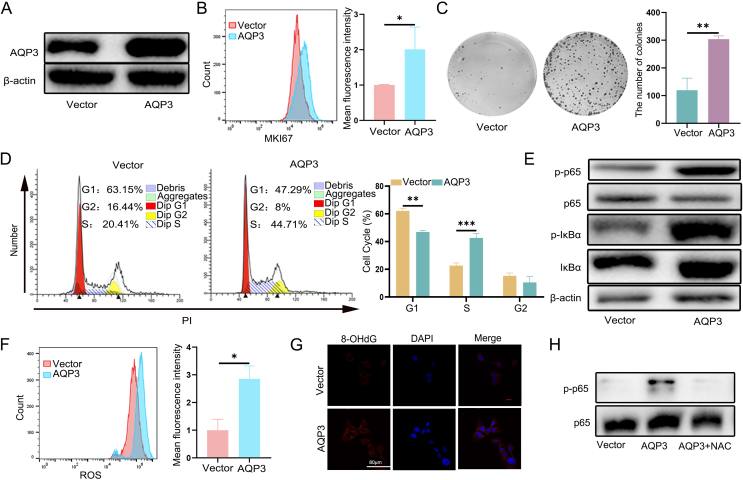# Aquaporin-3 promotes proliferation and inflammation in hepatocellular carcinoma

**DOI:** 10.1016/j.gendis.2023.06.004

**Published:** 2023-07-16

**Authors:** Yao Wang, Yalan Wu, Xueying Li, Weiwei Song, Fengling Zheng, Xiaoying Mo, Yi Luo, Yun Li, Song Chen, Huanhuan Luo

**Affiliations:** a School of Basic Medical Sciences, Guangzhou University of Chinese Medicine, Guangzhou, Guangdong 510006, China; b The School of Health, Guangzhou Vocational University of Science and Technology, Guangzhou, Guangdong 510555, China; c Zhongshan Hospital of Traditional Chinese Medicine, Zhongshan, Guangdong 528401, China; d The Journal Center, Guangzhou University of Chinese Medicine, Guangzhou, Guangdong 510006, China; e Jiangsu Vocational College of Medicine, Yancheng, Jiangsu 224005, China; f Science and Technology Innovation Center, Guangzhou University of Chinese Medicine, Guangzhou, Guangdong 510006, China; g State Key Laboratory of Dampness Syndrome of Chinese Medicine, Guangzhou, Guangdong 510006, China

Carcinogenesis is closely associated with inflammation. Studies have shown that lipopolysaccharides (LPS) can contribute to hepatocellular carcinoma (HCC) development by inducing IL-6 and TNF-α production from the hepatic progenitor cells.^1^ Aquaporin 3 (AQP3), a water channel protein, has recently been found to play a critical role in cancer-associated inflammation.^2^^,^^3^ However, it is still unclear whether AQP3 is involved in LPS-triggered proliferation and inflammation in HCC. In this current study, we found that AQP3 expression was negatively correlated with the overall survival rate of HCC patients and positively associated with MKI67 expression in human HCC patients. *In vitro* study demonstrated that AQP3 promoted LPS-induced cell proliferation, colony formation, and inflammation in HCC cell lines. *In vivo* xenograft model further demonstrated that AQP3 could promote tumor growth induced by LPS. In summary, the present study proved that AQP3 functioned as an oncogene and might serve as a promising target for cancer therapy.

We first investigated the correlation between AQP3 expression and survival in HCC cancer patients. Our analysis revealed a negative correlation between tumoral AQP3 expression and patients' survival rate by using the Tumor Immune Estimation Resource (TIMER) database (Fig. 1A). Furthermore, we found that AQP3 expression was also positively associated with MKI67 expression (Fig. S1A; supplementary material 1). To validate the results obtained from the public database, we evaluated the AQP3 expression by IHC staining with our own patient's cohort and assessed its relationship to patients' age, gender, cirrhosis, hepatitis B surface antigen (HBsAg), alpha-fetoprotein (AFP), hepatitis C virus (HCV), and tumor differentiation (Table S1). Semi-quantitative analysis of AQP3 expression showed that AQP3 is highly expressed in HCC tissues compared with control (non-HCC) tissues (Fig. 1B). Additionally, the positive area of DAB staining confirmed the positive correlation between AQP3 and MKI67 expression (Fig. 1C). Overall, these findings showed that AQP3 is associated with HCC proliferation and poor prognosis, indicating AQP3 may act as a potential oncogene in HCC.

**Figure 1:**
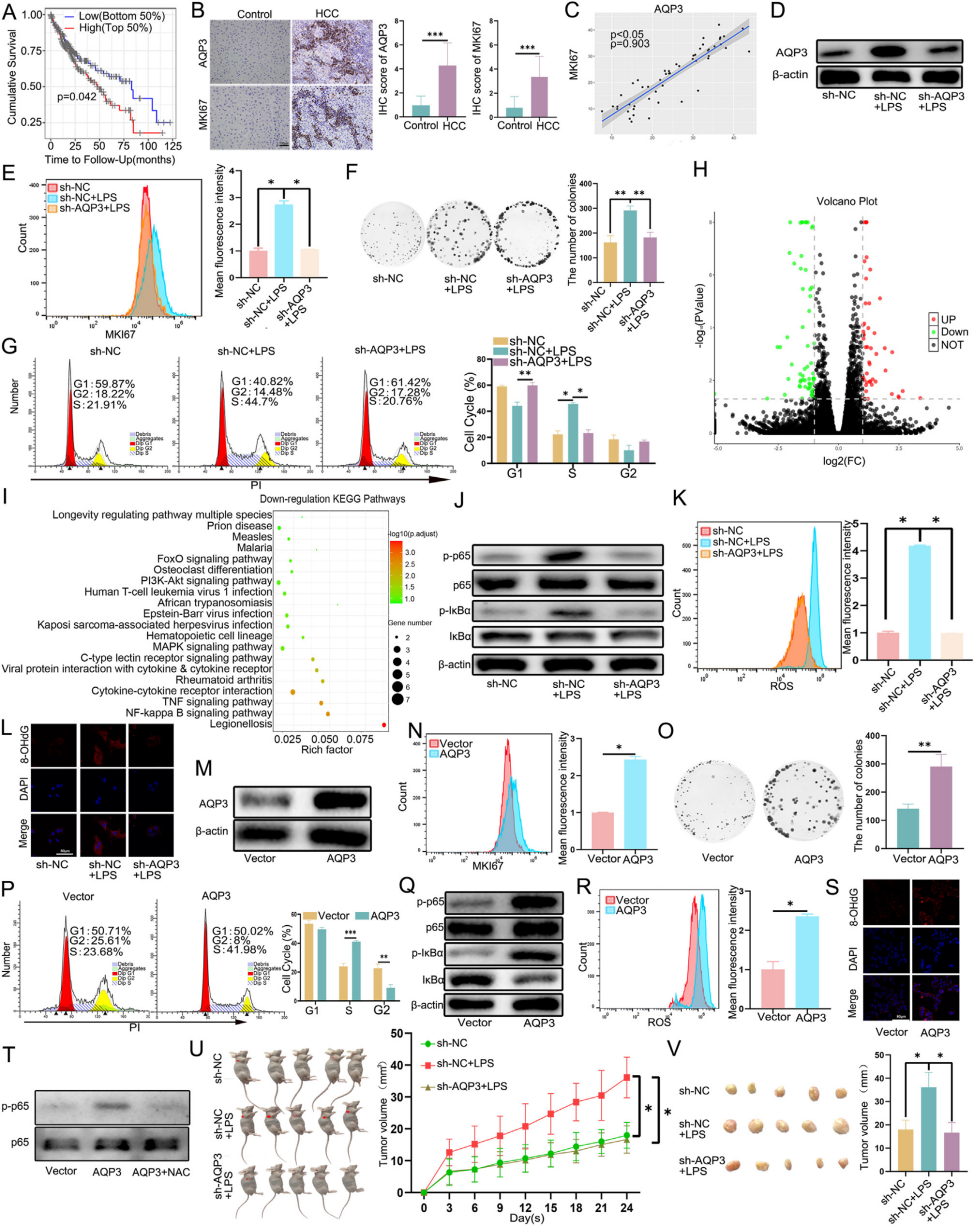
The correlation between AQP3 expression and survival in HCC cancer patients. **(A)** The Kaplan–Meier survival curve demonstrated the impact of AQP3 on the overall survival time of HCC patients (*n* = 370). **(B)** AQP3 and MKI67 expression were higher in HCC samples than in control samples. Scale bars: 100 μm. **(C)** The Spearman correlation coefficient was calculated to determine the relationship between relative protein expression levels of AQP3 and MKI67. **(D)** Western blot showed the protein expression of AQP3 in cells. sh-NC: cells transfected with control shRNA; sh-NC + LPS: sh-NC cells treated with LPS; sh-AQP3+LPS: cells transfected with sh-AQP3 and treated with LPS. **(E)** MKI67 assay was used to detect the proliferation capacity of AQP3 knockdown cells treated with LPS. **(F)** Plate colony experiment detected the role of AQP3 in the colony formation of HepG2 cells stimulated with LPS. **(G)** A cell cycle experiment was conducted to investigate the role of AQP3 in the increase of the S phase induced by LPS. **(H)** Volcano plot of DEGs in sh-AQP3+LPS *vs*. sh-NC + LPS (red for up-regulation and green for down-regulation). **(I)** Down-regulation KEGG pathway bubble chart of DEGs in sh-AQP3+LPS *vs*. sh-NC + LPS. **(J)** Western blot analysis was performed to examine the expression of p-p65, p65, p-IκBα, and IκBα proteins in HepG2 cells. **(K)** Intracellular ROS content in HepG2 cells. **(L)** Immunofluorescence analysis showed 8-OHdG expression in cells. Scale bar: 80 μm. **(M)** HepG2 cells were transfected with overexpression AQP3 (AQP3) or negative control (Vector) vector, and expression of AQP3 was analyzed by Western blot. Vector: cells transfected with empty plasmid without the sequence of AQP3 gene; AQP3: cells transfected with plasmid contained AQP3 construct gene. **(N)** MKI67 assay of HepG2 cells. **(O)** Colony formation assay. **(P)** Cell cycle distribution analysis in HepG2 cells after transfection with vector or AQP3. **(Q)** Western blot analysis was performed to assess the expression of the NF-B signaling pathway in vector and AQP3-overexpression cells. **(R)** The intracellular ROS content was measured in vector and AQP3 cells. **(S)** Immunofluorescence was used to visualize DNA oxidative damage in the cells. Scale bar: 80 μm. **(T)** The expression of p-p65 and p65 proteins in HepG2 cells after ROS elimination. **(U)** AQP3 knockdown suppressed tumor growth induced by LPS *in vivo.***(V)** The xenograft tumors with the treatment of LPS were significantly depressed by AQP3 depletion. The tumor volume of the xenografts was remarkably inhibited by AQP3 knockdown in xenografts treated with LPS (*n* = 5 in each group). ^∗^*P* < 0.05, ^∗∗^*P* < 0.01, ^∗∗∗^*P* < 0.001.

To investigate the effects of AQP3 on HCC cells treated with LPS, we knocked down the AQP3 expression in the HepG2 and Huh7 cells (Fig. 1D; Fig. S1B). We then assessed the effects of AQP3 on HCC cell proliferation using the MKI67 and colony formation assay. Our data revealed that AQP3 knockdown cells (sh-AQP3) had significantly lower proliferation compared with control cells (sh-NC) (*P* < 0.05; Fig. 1E, F; Fig. S1C, D). Furthermore, AQP3 knockdown led to a decrease in the percentage of cells in the S phase and an increase in the percentage of cells in the G1 phase in HepG2 cells after LPS stimulation (*P* < 0.05; Fig. 1G), suggesting the cell cycle was arrested at the G1 phase after AQP3 knockdown. Similar cell-cycle arrest was also observed in Huh7 cells with AQP3 knockdown (*P* < 0.05; Fig. S1E). These findings demonstrated that AQP3 knockdown can inhibit proliferation and lead to cell-cycle arrest in LPS-treated cells, indicating its potential as a therapeutic target for HCC treatment.

To dissect the mechanism of how AQP3 affects tumor cell proliferation, we performed RNA-seq with control or AQP3 knockdown HepG2 cells after LPS stimulation. Compared with control cells, we identified 109 differentially expressed genes (DEGs), including 46 up-regulated and 63 down-regulated genes (Fig. 1H; supplementary material 2) in AQP3 knockdown cells. Kyoto Encyclopedia of Genes and Genomes (KEGG) analysis revealed that legionellosis, PI3K-AKT, NF-κB, and TNF signaling pathways were altered by AQP3 knockdown (Fig. 1I). Western blot was used to validate RNA-Seq results and we discovered that AQP3 knockdown could suppress the activation of the NF-κB signal pathway induced by LPS in HepG2 cells (Fig. 1J; Fig. S1F). Previous studies have shown that reactive oxygen species (ROS) played a critical role in NF-κB activation and promote cell survival.^4^ Therefore, we measured the accumulation of ROS by flow cytometry and oxidative DNA damage by confocal microscopy (*P* < 0.05; Fig. 1K, L; Fig. S1G, H). Our results demonstrated that AQP3 knockdown inhibited the production of ROS induced by LPS. These findings suggest that AQP3 may play a crucial role in regulating the NF-κB signaling pathway through ROS production.

To investigate whether overexpression of AQP3 has an opposite effect to its knockdown, we overexpressed AQP3 in HepG2 and Huh7 cells (Fig. 1M; Fig. S2A) and examined the effects of AQP3 overexpression on cell proliferation, colony formation, cell cycle, NF-κB activation, and ROS production. The results showed that AQP3 promotes cell proliferation in both cell lines (*P* < 0.05; Fig. 1N; Fig. S2B), and the colony formation assay demonstrated that AQP3 overexpression increases the ability of HepG2 and Huh7 cells to form colonies (*P* < 0.05; Fig. 1O; Fig. S2C). Additionally, AQP3 overexpression caused an increase in the number of cells in the S phase, supporting our hypothesis (*P* < 0.05; Fig. 1P; Fig. S2D). Western blot analysis revealed that cells with AQP3 overexpression exhibited higher expression of NF-κB than vector cells (Fig. 1Q; Fig. S2E). We also assessed intracellular ROS levels by flow cytometry, and the results indicated that overexpression of AQP3 significantly increased the accumulation of intracellular ROS (*P* < 0.05; Fig. 1R; Fig. S2F). Additionally, we also found that AQP3 was associated with oxidative DNA damage (Fig. 1S; Fig. S2G). We further used N-acetylcysteine (NAC) to eliminate the ROS produced by overexpressed AQP3 and found a decrease in NF-κB expression, further demonstrating that AQP3 may activate the NF-κB signaling pathway through ROS (Fig. 1T; Fig. S2H).

To further explore the role of AQP3 on HCC growth *in vivo*, sh-AQP3 and sh-NC stably transfected with HepG2 cells were subcutaneously injected into the right flank of nude mice. The results showed that LPS stimulation significantly promoted tumor growth *in vivo*. Importantly, AQP3 knockdown prevented the LPS-induced increase in tumor growth (Fig. 1U, V). These findings suggest that AQP3 is involved in promoting HCC cell growth induced by LPS *in vivo* and that AQP3 knockdown can effectively inhibit this process.

In summary, the present results revealed that AQP3 may play an important role in proliferation, colony formation, and inflammation in HCC induced by LPS. Therefore, AQP3 may serve as a predictive biomarker and potential therapeutic target for HCC.

## Ethics declaration

The Ethics Committee of Zhongshan Hospital of Chinese Medicine approved this study, and informed consent was obtained from all patients. All experimental procedures of animals were approved by the Institutional Animal Care and Use Committee of Ruige Biotechnology.

## Conflict of interests

The authors have no competing interests to declare.

## Funding

This study was supported by grants from the National Natural Science Foundation of China (No. 82174244, 81973720), the Guangzhou Science and Technology Plan Project (Guangdong, China) (No. 201904010185), and the International Participant Application for Higher Education Track (China) (No. 202110572025).

## Figures and Tables

**figs1:**
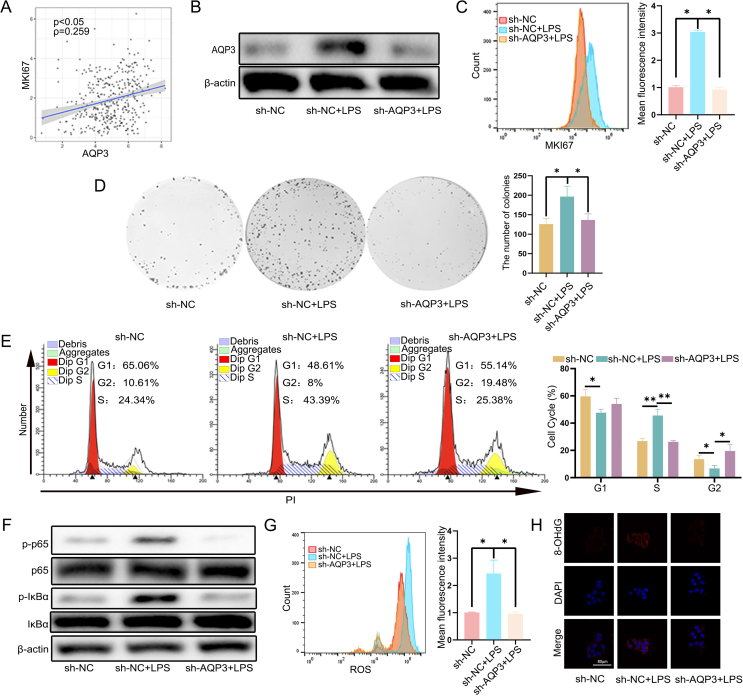


**figs2:**