# Supplementary material for: Aquaporin-3 promotes proliferation and inflammation in hepatocellular carcinoma
Source: Genes Dis. 2023 Jul 16;11(4):101029. doi: 10.1016/j.gendis.2023.06.004 (PMC10924172; doi:10.1016/j.gendis.2023.06.004)
Supplement: Multimedia component 1 [file mmc1.docx]

**Supplementary figure legend**

**Figure S1.** (A) The expression of AQP3 was positively correlated with MKI67 (n=371). (B) Western blot showed the protein expression of AQP3 in cells. (C) MKI67 assay was used to detect the proliferation capacity of AQP3 knockdown cells treated with LPS. (D) Plate colony experiment detect the role of AQP3 in colony formation of Huh7 cells stimulated with LPS. (E) Cell cycle experiment was used to show the role of AQP3 in the process of the increase of S phase induced by LPS. (F) Western blot analysis was performed to examine the expression of p-p65, p65, p-IκBα and IκBα proteins in Huh7 cells. (G) Intracellular ROS content in Huh7 cells. (H) Immunofluorescence analysis showed the expression of 8-OHdG in cells. Scale bar: 80 μm. *: *P* < 0.05, **: *P* < 0.01, ***: *P* < 0.001.

**Figure S2.** (A) Huh7 cells were transfected with overexpression AQP3(AQP3) or negative control (Vector) vector, and expression of AQP3 was analyzed by western blot. (B) MKI67 assay of Huh7 cells. (C) Colony formation assay. (D) Cell cycle distribution analysis in Huh7 cells after transfection with vector or AQP3. (E) Western blot analysis was performed to assess the expression of NF-ĸB signaling pathway in vector and AQP3-overexpressing cells. (F) The intracellular ROS content was measured in vector and AQP3 cells. (G) Immunofluorescence was used to visualize DNA oxidative damage in the cells. Scale bar: 80 μm. (H) The expression of p-p65 and p65 proteins in Huh7 cells after elimination ROS. *: *P* < 0.05, **: *P* < 0.01, ***: *P* < 0.001.
