# Supplementary material for: Aquaporin-3 promotes proliferation and inflammation in hepatocellular carcinoma
Source: Genes Dis. 2023 Jul 16;11(4):101029. doi: 10.1016/j.gendis.2023.06.004 (PMC10924172; doi:10.1016/j.gendis.2023.06.004)
Supplement: Multimedia component 2 [file mmc2.pdf]

Table1: Association of AQP3 expression with clinicopathological features of HCC patients.

Characteristics	Number of patients	AQP3 High expression	AQP3 Low expression	*P*
	51	29（56.5%）	22（43.5%）	
**Age(years)**				
≤50	33	19（58%）	14（42%）	NS/0.889
> 50	18	10（56%）	8（44%）	
**Gender**				
Male	39	23（59%）	16（41%）	NS/0.583
Female	12	6（50%）	6（50%）	
**Cirrhosis**				
Yes	21	15（71%）	6（29%）	NS/0.079
No	30	14（47%）	16（53%）	
**HBsAg**				
Positive	29	19（66%）	10（34%）	NS/0.152
Negative	22	10（45%）	12（54%）	
**AFP**				
Positive	27	20（74%）	7（26%）	0.008
Negative	24	9（37%）	15（63%）	
**HCV infection**				
Yes	2	2（100%）	0（0%）	NS/0.128
No	49	27（55%）	22（45%）	
**Tumor differentiation**				
High	8	4（50%）	4（50%）	NS/0.878
Moderate	15	10（67%）	5（33%）	
Poor	28	15（54%）	13（46%）	

Note: "NS" refers to that the differences among groups have no statistical significance.
