# Supplementary material for: Aquaporin-3 promotes proliferation and inflammation in hepatocellular carcinoma
Source: Genes Dis. 2023 Jul 16;11(4):101029. doi: 10.1016/j.gendis.2023.06.004 (PMC10924172; doi:10.1016/j.gendis.2023.06.004)
Supplement: Supplementary file 5 — Multimedia component 5 [file mmc5.docx]

Aquaporin-3 promotes proliferation and inflammation in hepatocellular carcinoma

**Materials and methods**

**Patients and tissue samples**

The Ethics Committee of Zhongshan Hospital of Chinese Medicine approved this study, and informed consent was obtained from all patients. All specimens were anonymized according to ethical and legal standards The retrospective study included 84 clinical samples, consisting of 51 cases of HCC and 33 cases of non-HCC samples (28 adjacent tissue and 5 intrahepatic bile duct stones), from patients who underwent curative liver resection at Zhongshan Hospital of Chinese Medicine. Paraffin-embedded tissues were retrieved from the tissue bank of the Department of Pathology at Zhongshan Hospital of Chinese Medicine. HCC diagnosis was based on the World Health Organization (WHO) criteria^1^ and tumor differentiation was defined according to the Edmondson grading system^2^. Liver function was assessed using the Child-Pugh scoring system^3^. The clinicopathological features of the 51 HCC patients are summarized in supplementary Table 1.

**Immunohistochemistry**

84 cases of 4% paraformaldehyde-fixed, paraffin-embedded tissue blocks were sectioned at 4 μm and underwent deparaffinization, hydration, endogenous peroxidase activity blocking, and antigen retrieval. The tissue sections were then incubated overnight at 4°C with antibodies against AQP3 (1:200, ab125219, Abcam, USA) and MKI67 (1:200, abs130135, absin, China). Subsequently, HRP-conjugated secondary antibodies were applied at 37°C for 1 hour, and DAB was used for visualization. IHC staining scores and percent positivity of AQP3 and MKI67 cells in hepatocytes were evaluated using the same methods as previous studies^4^.

**Transcriptome analyses**

The RNA samples were analyzed using an Agilent 2100 Bioanalyzer and RNA Nano 6000 Assay Kit (Agilent Technologies, CA, USA). The NEBNext® UltraTM RNA Library Prep Kit for Illumina® (NEB, USA) was utilized to prepare sequencing libraries, which were subsequently sequenced on an Illumina NovaSeq 6000 platform (Illumina). Significantly differentially expressed genes were identified using a P value threshold of less than 0.05. The read count data obtained from gene expression level analysis was used as the input data for gene expression differential analysis. DESeq2 was used for differential expression analysis of genes for samples with biological replicates, while edgeR software was used for experiments without biological replicates. RNA-seq differential gene analysis is a large-scale independent statistical hypothesis test that can lead to a high overall false positive rate. To account for this, a correction value was applied, with the default criteria for difference significance being padj less than 0.05 and |log2foldchange| greater than 1.

**Cell culture and treatment**

The HepG2 and Huh7 cells were purchased from National Collection of Authenticated Cell Cultures (Shanghai, China), and maintained at 37 °C in 5 % CO_2_ saturated humidity, in Dulbecco’s modified Eagle’s medium (DMEM) (GIBCO-BRL, New York, USA) supplemented with 10% fetal bovine serum (Gibco BRL). The concentration of LPS (L2880, Sigma-Aldrich, Germany) used to stimulating the cells was 400ng/ml.

**Over-expression and knockdown of AQP3**

Overexpression and knockdown lentiviruses for AQP3 (NM_004925) and control lentivirus were purchased from OBiO Corporation (Shanghai, China). Transfection was performed according to the manufacturer’s instructions and the MOI=10. Puromycin (2 μg/ml, Sigma) was used to select stable clones for at least 1 week. At the indicated time points, the cells were harvested for protein analysis as well as for other assays. And the results of PCR to detect the efficiency of transfecting were shown in supplementary material 4.

**Protein extraction and Western blot**

To detect related protein expression, cells were lysed with RIPA protein lysis buffer. The lysate was then centrifuged at 15,000 rpm for 30 min at 4°C, and the resulting supernatant was stored for later use. All procedures were conducted on ice. After SDS-PAGE separation, proteins were transferred and blocked with 5% BSA. Primary antibodies used included anti-AQP3, anti-p65, anti-p-p65, anti-IκBα, anti-p-IκBα, and anti-β-actin (1:1000; Cell Signaling Technology, USA). For detection, HRP-linked anti-rabbit antibody (1:5000; Cell Signaling Technology, USA) was used with the Tanon 5200 system (Shanghai, PR China). Band intensity was quantified using ImageJ software.

**Colony formation assay**

Cells were plated approximately 300 cells/well in 6-well plates with DMEM medium containing 10% FBS were cultured for 2-3 weeks. Colonies of cells were then counted after 4% paraformaldehyde was used to fix the cells for 10 min and crystal violet was used to stain the cells.

**Cell cycle analysis**

Cell cycle phases were assessed using flow cytometry analysis with PI staining. Initially, cells were grown in 6-well plates until they reached approximately 90% confluence. The cells were then collected via trypsinization without EDTA, washed twice with PBS, and gradually added 2.5 mL/sample of 70% ethanol while vortexing to loosen the pellet. After an overnight incubation at -20°C, the cells were washed twice with PBS and stain buffer to remove ethanol, followed by centrifugation and re-suspension in 500 μL PI for 15 minutes at room temperature. Finally, cells were re-suspended in 100 µL PBS and analyzed for cell cycle phases using a BD Accuri c5 flow cytometer. Data analysis was performed using ModFit LT software (Verity Software House, USA).

**MIK67 Assay**

After trypsinization, the cells were harvested via centrifugation at 500g for 3 minutes. Then, they were fixed with 100 μL of 4% paraformaldehyde at 4°C for 15 minutes and washed twice with 1X PBS. The cell pellet was re-suspended in 100 μL of 1X PBS and permeabilized with 90% ice-cold methanol for over 10 minutes. The cells were then washed with excess 1X PBS via centrifugation to remove methanol and incubated with an anti-MKI67 antibody (1:50) for 1 hour at room temperature. After two washes with 1X PBS, the cells were re-suspended in 200-500 µl of 1X PBS for flow cytometric analysis.

**ROS Assay**

An ROS assay kit (Beyotime, China) was used to measure ROS levels in cells. Based on the manufacturer’s instructions, HCC cells were incubated with 10μmol/L fluorescent DCFH-DA probe in cell culture medium at 37°C for 15 minutes. Cells were then washed 2 times by centrifugation with 1X PBS and resuspend in 200-500 µl 1X PBS prior to analysis using BD Accuri c5 flow cytometer.

**Confocal microscopy**

Cells were cultured in a 35-mm laser confocal Petri dish from Sorfa (Zhejiang, China) until they reached 60%–80% confluence. The following steps were used to treat the cells: 1) Fixing with 4% paraformaldehyde at room temperature for 30 min; 2) Washing with PBS three times; 3) Fixing with 0.1% Triton X-100 at 4°C for 10 min; 4) Washing with PBS three times; 5) Blocking with QuickBlock™ Blocking Buffer for Immunol Staining (Beyotime, Shanghai, PR China) at room temperature for 1h; 6) Incubation with anti-DNA/RNA damage antibody (ab62623) (1:200) overnight at 4°C; 7) Washing with PBS three times; 8) Incubation with goat polyclonal secondary antibody specific to goat anti-mouse IgG H&L(Alexa Fluor® 555) (1:200) (ab150114) at room temperature for 1h; 9) Washing with PBS three times; 10) Staining with DAPI for 10 min. Finally, the stained cells were imaged and analyzed using NIS-Elements Viewer 5.21 (Nikon, Japan).

**Tumor xenograft mouse model**

2 × 10^7^ cells were subcutaneously injected into the dorsal right flank of 6-week-old male Balb/c nude mice. LPS was dissolved in PBS and administered daily by subcutaneous injection. Tumor volumes were measured every three days using a vernier caliper. At the end of the experiment, mice were sacrificed and tumors were excised. All experimental procedures were approved by the Institutional Animal Care and Use Committee of Ruige Biotechnology.

**Statistical analysis**

Statistical analyses were performed using SPSS. The association between AQP3 expression and clinicopathological features in HCC patients was assessed using χ2 or Fisher's exact test. Data were presented as mean ± SD. Differences between two experimental groups were analyzed using Student's t-test. The correlation between AQP3 expression and MKI67 was evaluated using Spearman's correlation. Reported P values were two-sided, and statistical significance was set at p<0.05.

**Author contributions**

Yao Wang and Xueying Li: Conceptualization, Methodology, Software, Writing - Original Draft, and Writing - Review & Editing.Yalan Wu and Weiwei Song: Conceptualization, Methodology, Software, and Writing - Original Draft.Xiaoying Mo, Yi Luo, Fengling Zheng, and Yun Li: Methodology and Investigation.Song Chen and Huanhuan Luo: Funding Acquisition, Supervision, and Writing - Review & Editing.

**COMPETING INTERESTS**

The authors declare no competing interests.

**Funding statement**

The authors are grateful for the financial support provided by the National Natural Science Foundation of China (82174244, 81973720), the Guangzhou Science and Technology Plan Project (201904010185) and International Participant Application for Higher Education Track (202110572025).

**References:**

1. Sakamoto M. Pathology of early hepatocellular carcinoma. *Hepatology Research.* 2007;37:S135-S138.

2. Kulesza P, Torbenson M, Sheth S, Erozan YS, Ali SZ. Cytopathologic grading of hepatocellular carcinoma on fine‐needle aspiration. *Cancer Cytopathology: Interdisciplinary International Journal of the American Cancer Society.* 2004;102(4):247-252.

3. Tsoris A, Marlar CA. Use of the Child Pugh score in liver disease. 2019.

4. Wu G, Liu H, He H, et al. miR-372 down-regulates the oncogene ATAD2 to influence hepatocellular carcinoma proliferation and metastasis. *BMC cancer.* 2014;14:1-11.
